# Electroacupuncture stimulation enhances the permeability of the blood-brain barrier: A systematic review and meta-analysis of preclinical evidence and possible mechanisms

**DOI:** 10.1371/journal.pone.0298533

**Published:** 2024-03-27

**Authors:** Nuo Xu, Peng Gong, Shiting Xu, Yangyun Chen, Mengyuan Dai, Zhaoxing Jia, Xianming Lin

**Affiliations:** 1 The Third Clinical Medical College, Zhejiang Chinese Medical University, Hangzhou, China; 2 The Third Affiliated Hospital of Zhejiang Chinese Medical University, Hangzhou, China; Mayo Clinic Minnesota, UNITED STATES

## Abstract

An important cellular barrier to maintain the stability of the brain’s internal and external environment is the blood-brain barrier (BBB). It also prevents harmful substances from entering brain tissue through blood circulation while providing protection for the central nervous system. It should be noted, however, that the intact BBB can be a barrier to the transport of most drugs into the brain via the conventional route of administration, which can prevent them from reaching effective concentrations for the treatment of disorders affecting the central nervous system. Electroacupuncture stimulation has been shown to be effective at opening the BBB in a series of experimental studies. This study systematically analyzes the possibility and mechanism by which electroacupuncture opens the BBB. In PubMed, Web of Science, VIP Database, Wanfang Database, and the Chinese National Knowledge Infrastructure, papers have been published for nearly 22 years aimed at opening the BBB and its associated structures. A comparison of EB content between electroacupuncture and control was selected as the primary outcome. There were also results on vascular endothelial growth factor (VEGF), nerve growth factor (NGF), P-Glycoprotein (P-gp), Matrix Metalloproteinase 9 (MMP-9), and glial fibrillary acidic protein (GFAP). We utilized Review Manager software analysis to analyze correlations between studies with a view to exploring the mechanisms of similarity. Evans Blue infiltration forest plot: pooled effect size of 2.04, 95% CI: 1.21 to 2.87, P < 0.01. These results indicate that electroacupuncture significantly increases EB penetration across the BBB. Most studies have reported that GFAP, MMP-9, and VEGF were upregulated after treatment. P-gp expression decreased as well. Electroacupuncture can open the BBB, and the sparse-dense wave is currently the most effective electroacupuncture frequency for opening the BBB. VEGF plays an important role in opening the BBB. It is also important to regulate the expression of MMP-9 and GFAP and inhibit the expression of P-gp.

## 1 Background

The blood-brain barrier (BBB) is established through the interaction of vascular endothelial cells with astrocytes and pericytes, in conjunction with the basement membrane and various connexins. This barrier constitutes a dynamic system whose functionality is regulated by the central nervous system. The structure accountable for this adaptability are termed neurovascular units.

However, it also hinders the transport of most drugs into the brain, so it’s not able to reach the effective concentration of drugs for treating central nervous system diseases.

The most common method of opening the BBB is to use a combination of drugs such as hypertonic solutions, as well as nanopreparations, intracerebroventricular injection, lateral cerebral ventricular injection, Extracorporeal Shockwave Therapy, Electromagnetic Fields, etc [1]. These methods, however, are often limited in their use due to their own open technologies and means, and they are often difficult to implement. Aside from its beneficial effects, it is also known to cause serious adverse reactions, such as addictve behavior [2] and cytotoxicity [3]. In light of the current state of affairs, it is necessary to seek a new and alternative treatment option.

In Chinese medicine, acupuncture has been utilized for thousands of years as a topical therapy for a variety of ailments due to its safety and potential effectiveness [4]. EA is a method of applying a small current wave close to human bioelectricity to the needle after deqi in order to increase efficacy. In clinical research reports, EA has been proven to have neuroprotective and neuroregenerative properties, as well as significant increases in cerebral blood flow, regulation of microcirculation and oxygen metabolism in brain tissue, inhibition of inflammatory responses, and reduction of apoptosis [5, 6].

Currently, there exist systematic studies on the effects of electroacupuncture to promote the opening of the BBB, and several potential mechanisms have been postulated. As an illustration, electroacupuncture has the capacity to activate the sensory distribution region associated with the trigeminal nerve, thereby triggering the release of neurotransmitters like glutamate. This process leads to heightened neural activity within the cerebral cortex and elicits the opening effect of the blood-brain barrier [7]. On the other hand, electroacupuncture affects glial membrane barrier tight junction proteins. This intervention decreases ZO-1 and occludin expression, widening the intercellular space between vascular endothelial cells and increasing blood-brain barrier permeability [8]. Electroacupuncture can also affect the blood-brain barrier permeability by altering the gene expression of relevant transporters, such as solute carrier (SLC) transporters and ATP binding box (ABC) transporters, on endothelial cells [9]. Despite an increasing body of research on acupuncture’s ability to open the BBB, no systematic review has been conducted to examine the proven effects of electroacupuncture in preclinical research. The aim of this study is to conclude the role and mechanism of electroacupuncture therapy in opening the BBB and to provide a reference for subsequent clinical treatment.

## 2 Methods

### 2.1 Review process

A systematic review and meta-analysis of the literature was presented in compliance with PRISMA, the Preferred Reporting Items for Systematic Reviews and Meta-Analyses [10]. Review authors (NX and ZJ) initially screened title and abstract independently, and once shortlisted, the full-text manuscripts were independently screened for final inclusion.

### 2.2 Search strategy

The following electronic reference databases were searched: PubMed, Web of Science, WanFang Database, China National Knowledge Infrastructure, and VIP Database.

The search terms were shown: (((((((Blood-Brain Barrier[MeSH Terms]) OR ((Blood-Brain) AND (Barrier))) OR (((Matrix Metalloproteinases[MeSH Terms]) OR (Matrix Metalloproteinase 9[MeSH Terms])) OR (Matrix Metalloproteinase 2[MeSH Terms]))) OR ((Vascular Endothelial Growth Factor A[MeSH Terms]) OR (Vascular Endothelial Growth Factors[MeSH Terms]))) OR ((((((Cyclic GMP-Dependent Protein Kinases[MeSH Terms]) OR (Tight Junction Proteins[MeSH Terms])) OR (Claudins[MeSH Terms])) OR (Junctional Adhesion Molecules[MeSH Terms])) OR (Occludin[MeSH Terms])) OR (Zonula Occludens Proteins[MeSH Terms]))) OR ((Astrocytes[MeSH Terms]) OR (Cell Adhesion Molecules[MeSH Terms]))) OR (Albumins[MeSH Terms])) AND ((Electroacupuncture[MeSH Terms]) OR (Electroacupuncture)). Publication date until December 2023. All searches were limited to animal studies. There were no language restrictions as long as English references are provided.

### 2.3 Inclusion and exclusion criteria

Firstly, we included controlled experimental studies evaluating the effects of electroacupuncture stimulation on the BBB in rats. To avoid bias, these inclusion criteria were predetermined: (1) all animal models with brain diseases in rats. Basic characteristics, such as sex, age, weight, species, are not restricted; (2) electroacupuncture was administered to the experimental group, whereas the controls received a vehicle, saline, acupuncture or positive control drug or no treatment; (3) original articles with a separate control group. The treatment of electroacupuncture was carried out on the rat model regardless of the frequency, acupuncture point, or duration of the treatment. These outcomes included Evans Blue, nerve growth factor, glial fibrillary acidic protein, matrix metalloproteinase, phosphoglycoprotein, and vascular endothelial growth factors.

There were specified exclusion criteria: (1) in vitro studies, ex-vivo studies, human studies, and transgenic animal studies; (2) No electroacupuncture treatment, administration of electroacupuncture together with other drugs, electroacupuncture pre-stimulation; (3) case studies, cross-over studies, ex vivo studies, in vitro studies, human studies, transgenic animal studies, reviews, conference abstracts, systematic reviews, or meta-analyses; (4) studies without a separate control group; (5) studies with no quantitative data or results not presented in the form of graphs, charts, etc.

### 2.4 Data extraction

The information was collected from studies published in recent years using predesigned forms to obtain essential characteristics of the research. The data included the name of the author, publish date, country where the experiment was conducted, animal strain, sex, body weight, age, type of disease model, acupuncture points, electroacupuncture frequency, treatment schedule, and main outcomes. Main outcomes include vascular endothelial growth factor, nerve growth factor, P-Glycoprotein, Matrix Metalloproteinases, glial fibrillary acidic protein and other factors that affect the opening of the BBB. In each review, data were extracted from the literature by two independent reviewers. As a first step, we extracted accurate continuous values data from tables, text, and graphs. Due to the fact that the outcome measure is a continuous variable, we only extracted the mean and standard deviation. In the absence of these, we extracted data from graphs using GetData Graph Digitizer 2.20. A maximum of two attempts was made to contact authors when data were not supplied or uncertain. When an outcome was measured at more than one time point, data was included from all time points. The differences in opinion were negotiated and extracted once again. Any differences between them on the qualifications for a particular study were resolved through discussion with a third-independent review author.

### 2.5 Publication bias

Using the Systematic Review Centre for Laboratory Animal Experimentation’s (SYRCLE) risk of bias tool, two trained reviewers independently examined studies for bias [11]. Using the scale, 10 domains are classified as "Yes", "Unclear", or "No" based on the information provided in the publications.

### 2.6 Statistical analysis

Due to the differing scales used by most studies, we used the standardized mean difference (SMD) with 95% confidence intervals (CI). And statistical heterogeneity was evaluated by I-squared (I²) and Cochran’s Q statistics (Chi-square). The heterogeneity and variability among studies made random effects models the mainstay. Probabilities less than 0.05 were considered statistically significant. Subgroup analysis was used when heterogeneity is excessive. Review Manager 5.4 software was used for data analysis. As a final step, sensitivity analysis with article-by-article exclusion was conducted to test the robustness of the analysis.

## 3 Results

### 3.1 Study selection

**Fig 1:**
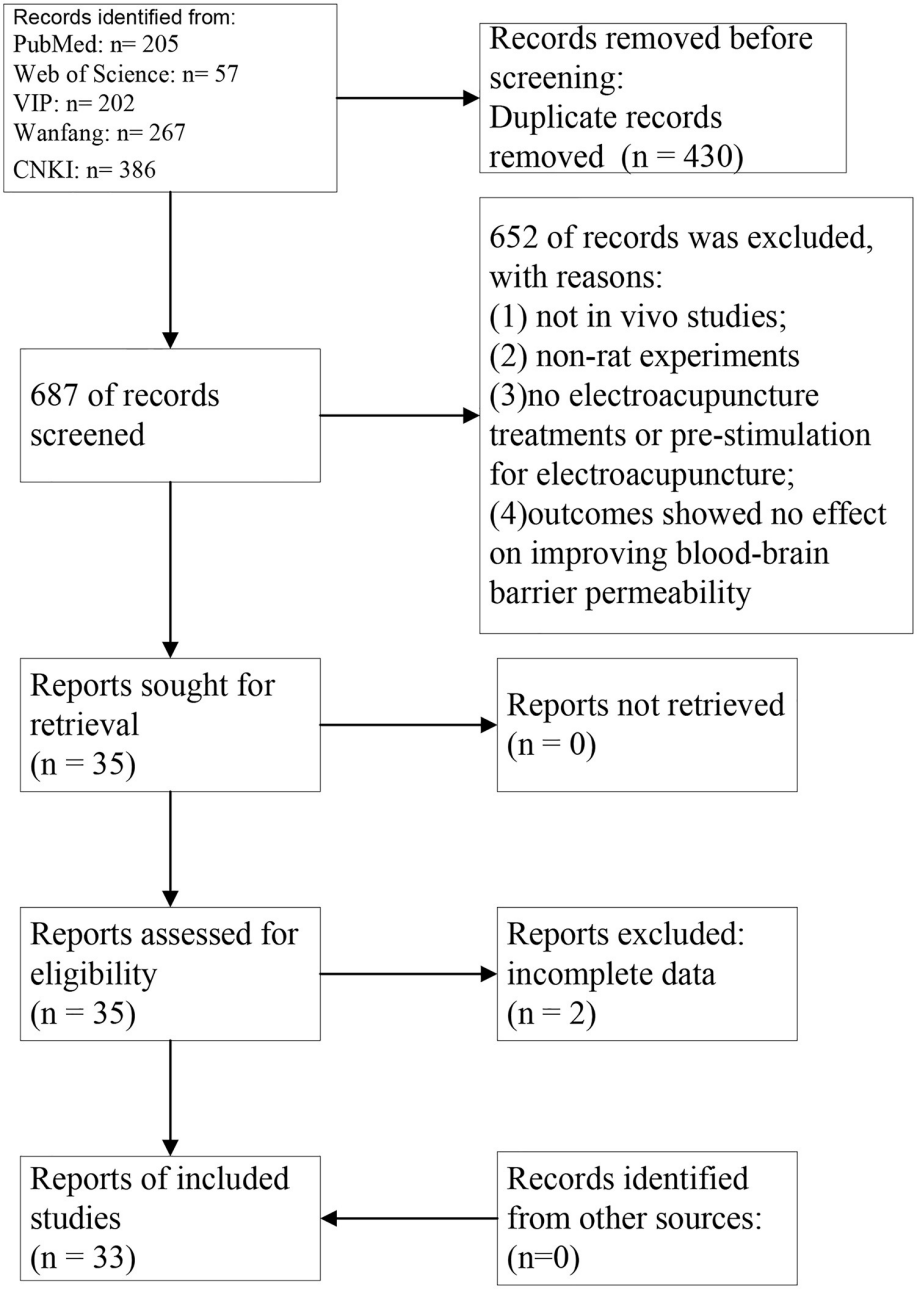
The PRISMA flow chart illustrates the selection process for systematic reviews.

According to our search, 1117 articles were retrieved, of which 430 were removed due to duplicate content. After reviewing the titles and abstracts, 687 studies were further removed for the following reasons: (1) not in vivo studies; (2) non-rat experiments; (3) no electroacupuncture treatments or pre-stimulation for electroacupuncture; (4) Results was reduced the permeability of the blood-brain barrier.; These articles were excluded from further analysis. In total, we included 33 studies [8, 12–43] in the fifinal analysis. In this study, the PRISMA diagram represents the inclusion and exclusion process (Fig 1), (S1 Checklist).

### 3.2 Study characteristics

Thirty-three studies analyzed 1188 rats, of which 689 were included in the experimental group and 499 in the control group. Eight studies were published in English and the rest in Chinese. In 14 studies [13, 14, 18, 19, 22, 23, 26, 28, 30, 32, 34–36, 40], continuous waves were used in the electroacupuncture group and sparse-dense waves were used in the electroacupuncture group in 18 studies [12, 15–17, 20, 21, 24, 25, 27, 29, 31, 33, 37–39, 41–43]. Additionally, in one study [8], both continuous and sparse-dense waves were used. Literatures included in the review were generally evaluated on the basis of established selection criteria, and they generally demonstrated the following: all animal subjects were rats, and electroacupuncture was administered to animals in the experimental group.

**Fig 2:**
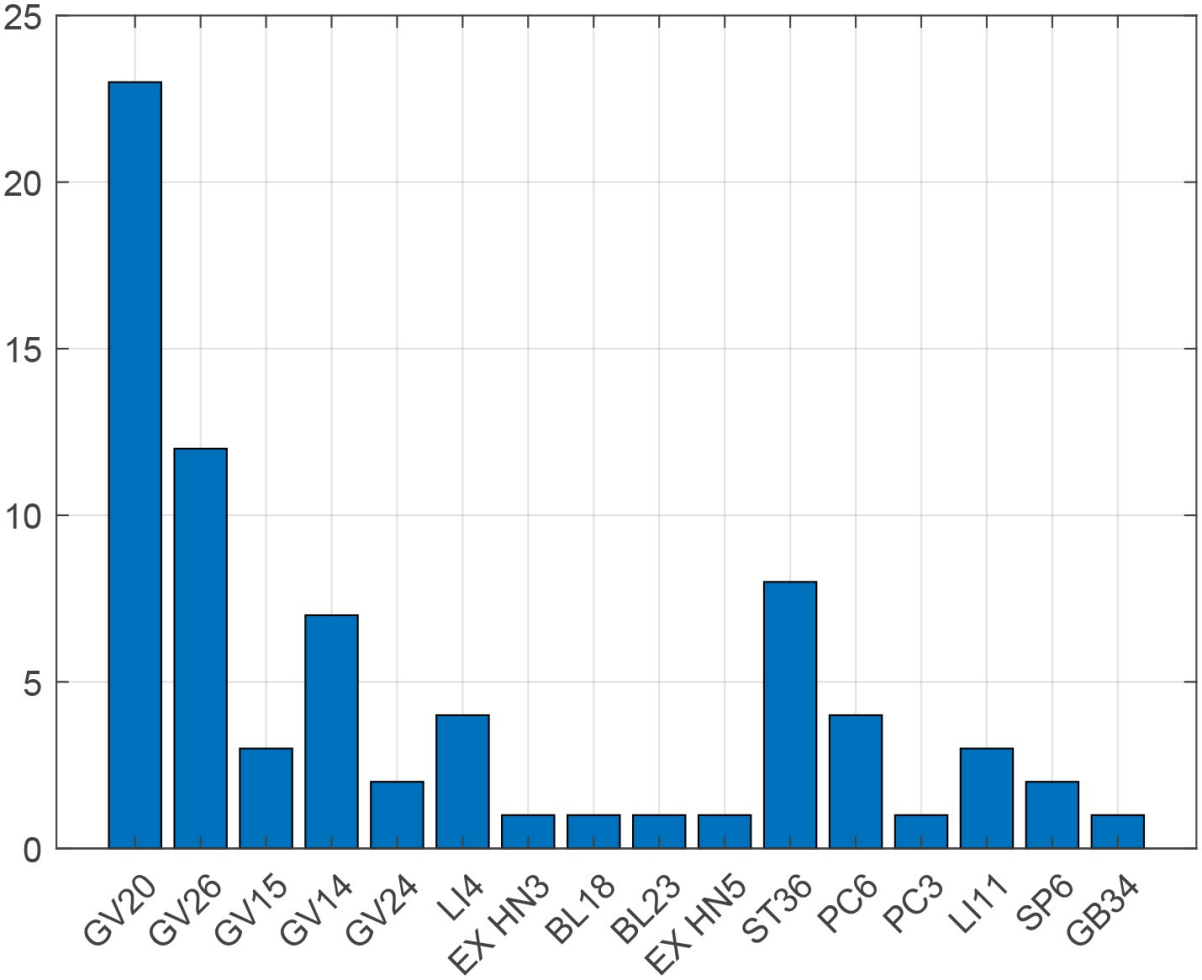
Frequency statistics of acupuncture points.

Based on the GSPC and STRICTA lists, we believe that acupoints, electroacupuncture frequency, treatment regimen, and sampling time are also factors that influence the effect size of the final outcome (Table 1). Fig 2 shows the main points of electroacupuncture stimulation.(Fig 2).

**Table 1:** Characteristics of the included studies.

Study	Study Location	Rats	Weight(g)	Model	Acupoints	Electroacupuncture Frequency	Treatment regimen	sampling time	Outcome Measures
Zhao et al., 2019	China	male, Sprague Dawley	275±25	MCAO(R)	GV20, GV26	Sparse-dense Wave, 2/15 Hz, 2/30 Hz, 2/50 Hz, 2/100 Hz	2 mA, 8 min, treated once	3 weeks after sugery	EB, NGF
Zhang et al., 2018	China	male, Sprague Dawley	250±20	MCAO	GV20, GV26	Continuous Wave, 15 Hz, 30 Hz, 50 Hz, 100 Hz	2 mA, 8 min, treated once	after BBB recovery	EB, P-gp
Lin et al., 2002	China	NR, Sprague Dawley	280±32	Normal	GV20, GV15	Continuous Wave, 50 Hz	5 mA, 15 min, treated twice	NR	EB
Cai et al., 2015	China	male, Wistar	250–300	VCI	GV20, GV14, GV24, GV26	Sparse-dense Wave, 2/20Hz	1–2 mA, 20 min, once a day for 20 days	4 days after surgery	VEGF
Chen et al., 2017	China	male, Sprague Dawley	220–250	MCAO	BL18, BL23	Sparse-dense Wave, 10/50 Hz	1–3 mA, 30 min, once a day for 3, 14 or 21 days	2 hours after surgery	VEGF, CD31+
Lei et al., 2019	China	male, Wistar	200–350	MCAO	GV20, EX HN5	Sparse-dense Wave, 5–30 HZ	30 min, once a day for 3, 7 or 14 days	NR	NGF, VEGF
Li et al., 2014	China	NR, Wistar	180–200	MCAO	GV26	Continuous Wave, 15 Hz	0.1 mA, 20 min, 3 h, 6 h, 12 h, 24 h, 3 days, 7 days, 12 days	immediately after sugery	VEGF
Liu et al., 2016	China	male, Sprague Dawley	200±20	MCAO	GV20, ST36(L)	Continuous Wave, 2 Hz	2 V, 30 min, once a day for 14 days	NR	VEGF
Ma et al., 2013	China	male, Wistar	250–300	MCAO(R)	LI4(bilateral)	Sparse-dense Wave, 40/60 Hz	1.5 V, 15 min, once a day for 7 days	45 min after surgery	VEGF
Mao et al., 2012	China	NR, Sprague Dawley	200±20	MCAO	GV20, GV26, ST36(bilateral)	Sparse-dense Wave, 2/15 Hz	1 mA, once a day for 1, 2, 4 or 8 days	1 hours after reperfusion	VEGF
Miu et al., 2014	China	male, Sprague Dawley	200±20	MCAO	GV20, ST36(NR)	Continuous Wave, 2 Hz	3 V, 30 min, once a day for two weeks	2 weeks after surgery	VEGF
Pan et al., 2012	China	male, Sprague Dawley	220–250	MCAO	LI4, PC6, PC3, LI11(unilatera)	Continuous Wave, 20 Hz	2–4 V, 30 min, treated once	6, 24, 48 or 72 hours after sugery	VEGF
Qian et al., 2014	China	male, Wistar	200±20	VD	GV20, GV14, GV24, GV26	Sparse-dense Wave, NR	20 min, once a day for 30 days, rest 2 days between 10 days	NR	VEGF
Sun et al., 2015	China	male, Sprague Dawley	240–260	MCAO(L)	LI11(R), LI4(R), PC6(R), ST36(R), SP6(R)	Sparse-dense Wave, 4/20 Hz	2 V, 0.5 mA, 20 min, once a day	24 hours after surgery	VEGF
Wan et al., 2010	China	male, Sprague Dawley	90–120	MCAO	GV20, GV14	Continuous Wave, 3 Hz	3V, 15 min, once a day for 1, 7 or 14 days	NR	VEGF
Wu et al., 2018	China	male, Sprague Dawley	180–220	ICH	ST36(bilateral)	Sparse-dense Wave, 2–15 Hz	2 mA, 20 min, once a day for 3, 7 or 14 days	NR	VEGF
Xiao et al., 2011	China	male, Sprague Dawley	250–300	MCAO(L)	ST36(NR), LI11	Sparse-dense Wave, 15 Hz, 30 Hz, 100 Hz	60–80 Ua, 30 min, once a day for 5 days	NR	GFAP
Xu et al., 2010	China	male, Sprague Dawley	280–310	MCAO	GV20, ST36(NR)	Sparse-dense Wave, 2–100 Hz	2 mA, 20 min, once a day for 72 or 96 hours	NR	MMP-9
Yu et al., 2013	China	NR, Sprague Dawley	180–220	MDD	GV20, GV15	Continuous Wave, 2 Hz	1 mA, 20 min, once a day for 14 days	8 days after surgery	P-gp
Zan et al., 2019	China	male, Sprague Dawley	300±20	MCAO	GV20, GV26, PC6(bilateral)	Sparse-dense Wave, 2/20 Hz	3–5 V, 20 min, once a day for 14 days	4 hours after surgery	VEGF, CD34+
Zhang et al., 2013	China	male, Sprague Dawley	180–220	MCAO(R)	GV20, GV14	Continuous Wave, 2 Hz	3 V, 30 min, once a day for 5 weeks	7 days after surgery	VEGF
Zhang et al., 2013	China	male, Sprague Dawley	250±50	MCAO(R)	GV20, GV14	Sparse-dense Wave, 2/30 Hz	2 mA, 2–4 V, 30 min, continued for 14 days	24 hours after surgery	VEGF
Zhao et al., 2018	China	male, Wistar	280–320	PSD	GV20, GV14	Continuous Wave, 50 Hz	2.0 rflA, 20 min, once a day for 15 days	NR	VEGF
Zhou et al., 2007	China	male, Sprague Dawley	220±20	MCAO	GV20, GV26, ST36(bilateral)	Sparse-dense Wave, 2/15 Hz	1 mA, 30 min, once a day for 8 days, treated once before analysis, total 9 sessions	8 days after reperfusion	VEGF
Cheng et al., 2009	China	male, Sprague Dawley	250–280	MCAO	GV20, GV26	Continuous Wave, 4 Hz	2 mA, 30 min, continued for 3 days	2 hours after surgery	NGF
Yao et al., 2021	China	male, Sprague Dawley	200±20g	CUMS	GV20, EX HN3	Continuous Wave, 2 Hz	0.6 mA, 30 min, once a day for 14 days	from day 22 to day 35	GFAP
Lu et al., 2013	China	NR, Sprague Dawley	250–300	MCAO	LI4	Sparse-dense Wave, 40/60 Hz	1 mA, 15 min, once a day for 3 or 7 days	1 hours after reperfusion	MMP-9
Han et al., 2010	China	male, Wistar	200±20	MCAO	GV20, GV14	Continuous Wave, 20 Hz	1–2 mA, 30 min, once a day for 7, 14 or 28 days	the day after sugery	GFAP
Lin et al., 2009	China	male, Sprague Dawley	200–220	VD	GV20, GV15	Continuous Wave, 100 Hz	2 mA, 20 min, treated once	21 days after sugery	NGF
Zhao et al., 2022	China	male, Sprague Dawley	250–270	MCAO(R)	GV20, GV26	Sparse-dense Wave, 2/100 Hz, 6 sec on and 6 sec off	2 mA, 40 min, treated once	3 weeks after sugery	NGF
Zhang et al., 2020	China	male, Sprague Dawley	NR	Normal	GV20, GV26	Continuous Wave, 2 Hz, 15 Hz, 30 Hz, 50 Hz, 100 Hz	5 min, 15 min, 40 min, 1 h, 2 h, 4 h, 5 h, 12 h	NR	EB, ZO-1, Claudin-5, GFAP
						Sparse-dense Wave, 2/50 Hz, 2/100 Hz			
Fu et al., 2023	China	male, Sprague Dawley	220±20	MACO(R)	GB34(bilateral)	Sparse-dense Wave, 2/100 Hz	2-4V, 20 min, once a day for 14 days	NR	VEGF
Zhang et al., 2022	China	male, Wistar	200±20	MACO(R)	GV26, PC6, SP6	Sparse-dense Wave, 2/15 Hz	1 mA, 90 min, once a day for 7 days	NR	VEGF

**Note:** CUMS:the chronic unpredictable mild stress, NR: not reported, MCAO:middle cerebral artery occlusion, PSD:poststroke depression, ICH:intracerebral hemorrhage, VD:vascular dementia, VCI:vascularcognitive impairment, R:right, L:left, MDD:Major Depressive Disorder, GV20:Baihui, GV26:Shuigou, GV15:Yamen, GV14:Dazhui, GV24:Shenting, LI4:Hegu, EX HN3:Yintang, BL18:Ganshu, BL23:Shenshu, EX HN5:Taiyang, ST36:Zusanli, PC6:Neiguan, PC3:Quze, LI11:Quchi, SP6:Sanyinjiao, GB34: Yanglingquan, EB:Evans Blue, NGF:nerve growth factor, VEGF:Vascular Endothelial Growth Factors, GFAP:glial fibrillary acidic protein, MMP-9:Matrix Metalloproteinase 9, P-gp:P-Glycoprotein, ZO-1:Zonula Occludens-1 Protein.

### 3.3 Publication bias

Nine studies [12, 16, 18, 25, 26, 38, 39, 30, 43] clearly stated their specific randomization methods. There are no publications that mention the implementation of allocation concealment and blinding. In all studies, the sex and body weight of the control and experimental groups of animals are mentioned as being similar. In addition, 14 publications [8, 12, 13, 16, 18, 21, 23, 27, 29, 30, 34, 41–43] described animals in the experimental and control groups being housed under the same conditions. One study [36] adequately explained incomplete data and used appropriate methods for estimation. No publication explicitly mentions whether animals were randomly selected for outcome analysis or whether their results were reported selectively.

### 3.4 Meta-analysis and results

#### 3.4.1 Effects on the EB content

**Fig 3:**
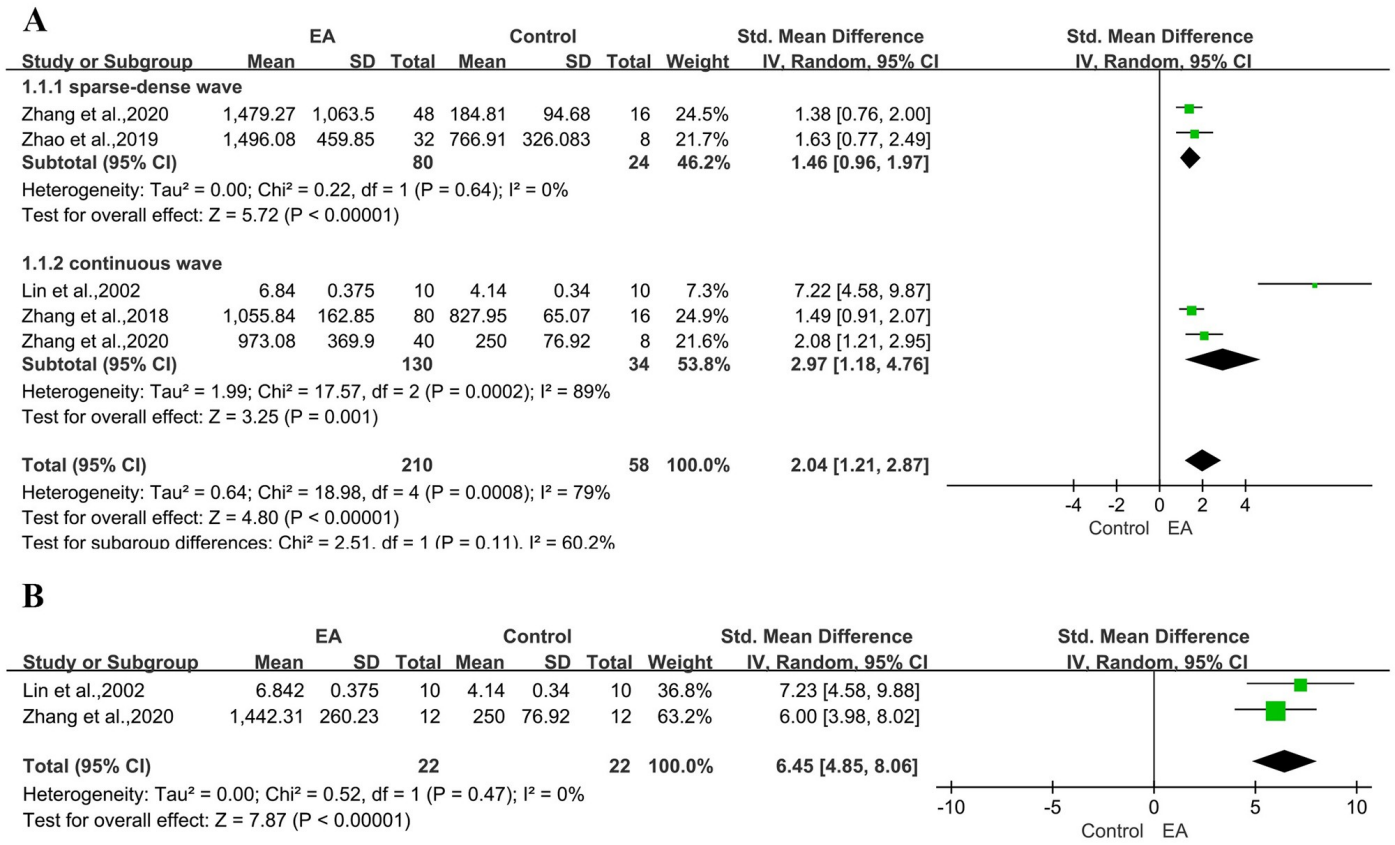
Forest plot of increased EB content in the brain from different studies. (A) Electroacupuncture frequency; (B) Physiological and pathological blood-brain barrier.

A combined analysis of four studies [8, 12–14] showed significant heterogeneity among studies(n = 268, SMD: 2.04, 95% CI: 1.21 to 2.87, P < 0.01). We judged the obvious heterogeneity to be due to the digital divide in electroacupuncture stimulation frequency and animal disease models across studies. According to our subgroup analysis, electroacupuncture frequency was not a significant source of the heterogeneity(Fig 3). Moreover, using healthy rats as the baseline condition, electroacupuncture can promote EB entry into the brain through the physiological and pathological BBB (Fig 3).

#### 3.4.2 Effects on the GFAP

A study [34] reported that EA therapy could reduce depression manifestations in CUMS rats, and the concentration of GFAP protein in their brains as well as the mean optical density of GFAP-immunoreactive astrocytes (GFAP-ir astrocytes) could be increased (n = 20, P < 0.05). According to the study [35], the expression of GFAP was increased in the EA group after 7 days, 14 days, and 28 days as compared to the control group. One study [38] also found that electroacupuncture at 15 Hz and 30 Hz improved the number of GFAP-positive astrocytes and their optical density, and also improved the number of cells in the ipsilateral proximal parietal cortex (n = 18, P < 0.01).

#### 3.4.3 Effects on the matrix metalloproteinases

**Fig 4:**
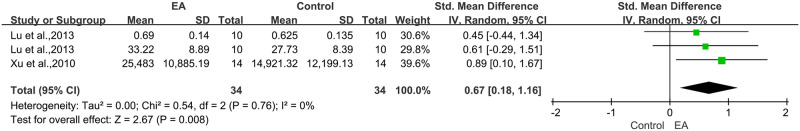
Forest plot of upregulated MMP-9 expression from different studies.

A combined analysis of two studies with three comparisons [39, 41] showed a significant effect of EA on stimulating positive cells and mRNA expression of MMP-9 compared with the control group. An effect size of statistically significant was found for one study [39], which concluded that the MMP-9 mRNA was highly expressed in the EA group when the duration reached 72 h and 96 h in contrast to the model group, where it peaked at 48 h and 144 h (Fig 4).

#### 3.4.4 Effects on NGF

**Fig 5:**
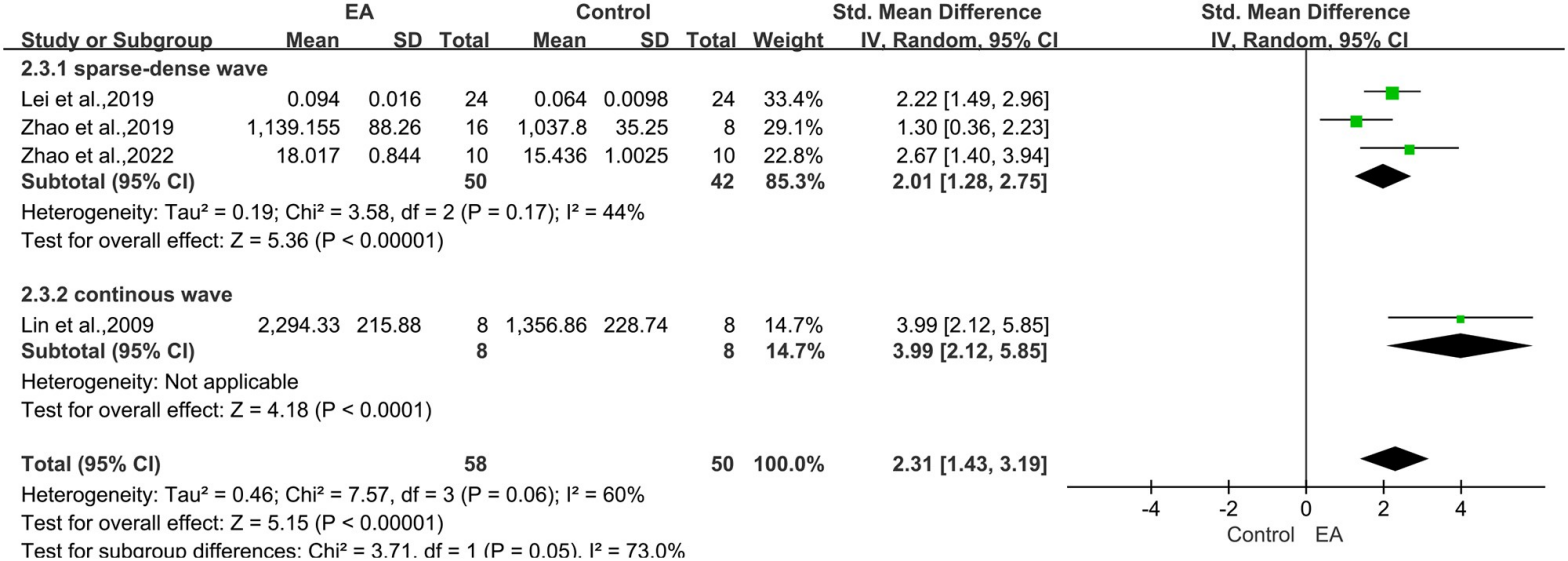
Forest plot of increasing NGF levels in the brain from different studies.

*NGF content*. Meta-analysis of 4 studies [12, 17, 36, 37] showed that the stimulation of electroacupuncture was significant for inducing NGF into the brain compared with the control groups (n = 108, SMD 2.31, 95% CI: 1.43 to 3.19, P < 0.01). Due to the moderate heterogeneity between studies (Chi-square = 7.57, df = 3, P = 0.06, I² = 60%), we performed the subgroup analysis in accordance with the electroacupuncture frequency, resulting in a more meaningful conclusion (Fig 5).

*Modified Neurological Severity Score (mNSS)*. Cheng et al. (2009) showed that mNSS was lesser in the IN group administered with NGF and EA, as compared to the IN group administered with NGF only (n = 24, P < 0.05).

*Others*. A study [37] reported that FITC-NGF relative fluorescence intensity in the control-EA group was greater than that of the control, as did the mean positive cell rate, which indicates that EA is capable of opening the physiological BBB in the prefrontal cortex. (n = 20, p < 0.01).

#### 3.4.5 Effects on P-gp

**Fig 6:**
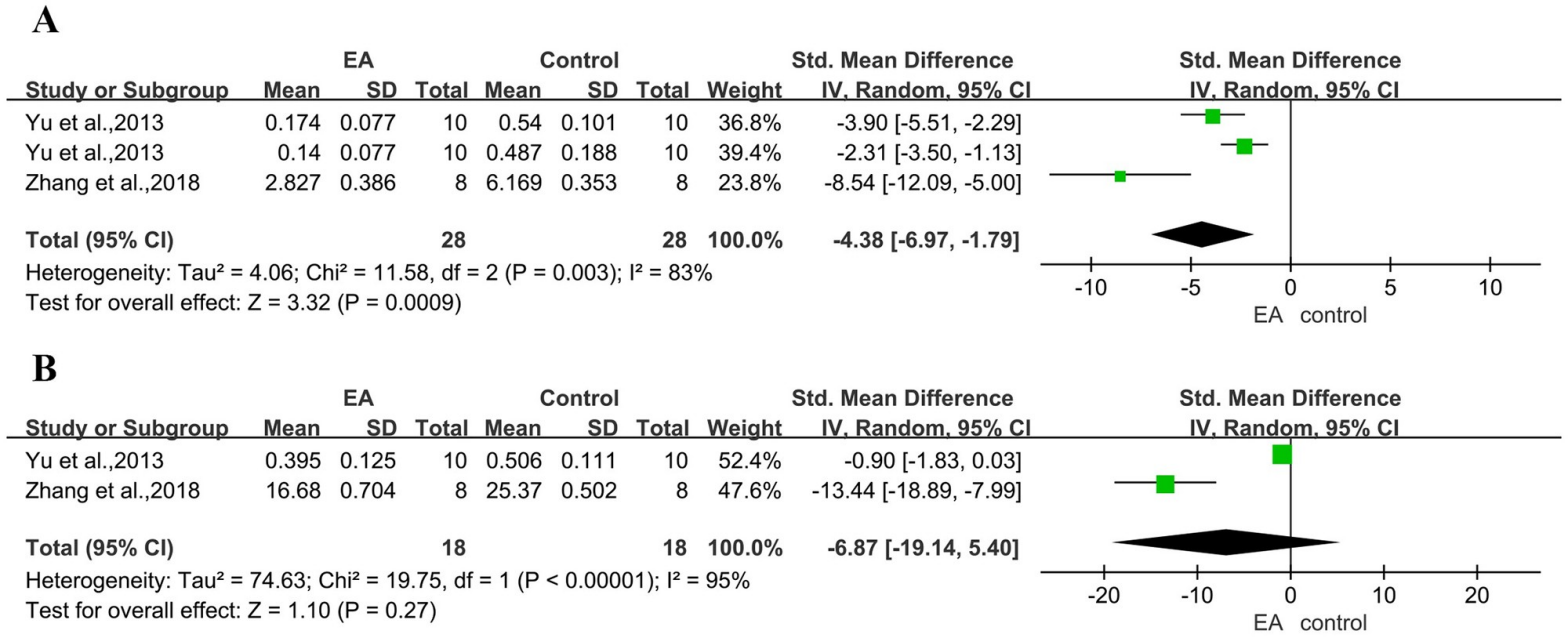
Forest plot of inhibition of P-gp levels from different studies. (A) mdr1a mRNA, (B) mdr1b mRNA.

A combined analysis of two studies with four comparisons [13, 28] showed the conclusion of electroacupuncture stimulation for reducing the mRNA expression of P-gp compared in the cerebral cortex region with control groups (Fig 6). We believe that the high heterogeneity is due to disease model and methodological differences in rats in different studies. Meanwhile, the results of the study [28] on mdr1b mRNA expression were not deemed positive.

#### 3.4.6 Effects on VEGF

*VEGF mRNA*. A combined analysis of 17 studies with 21 comparisons [15–27, 29–33, 42, 43] shows high heterogeneity between studies (n = 620, SMD: 2.27, 95% CI 1.59 to 2.95, P < 0.01; Heterogeneity: Chi-square = 189.79, df = 18, P <0.00001, I² = 91%) S1 Fig. There was a statistically significant effect size in each experiment, except for one study [19, 42], which indicates that the treatment significantly enhances the production of vascular endothelial growth factor.

**Fig 7:**
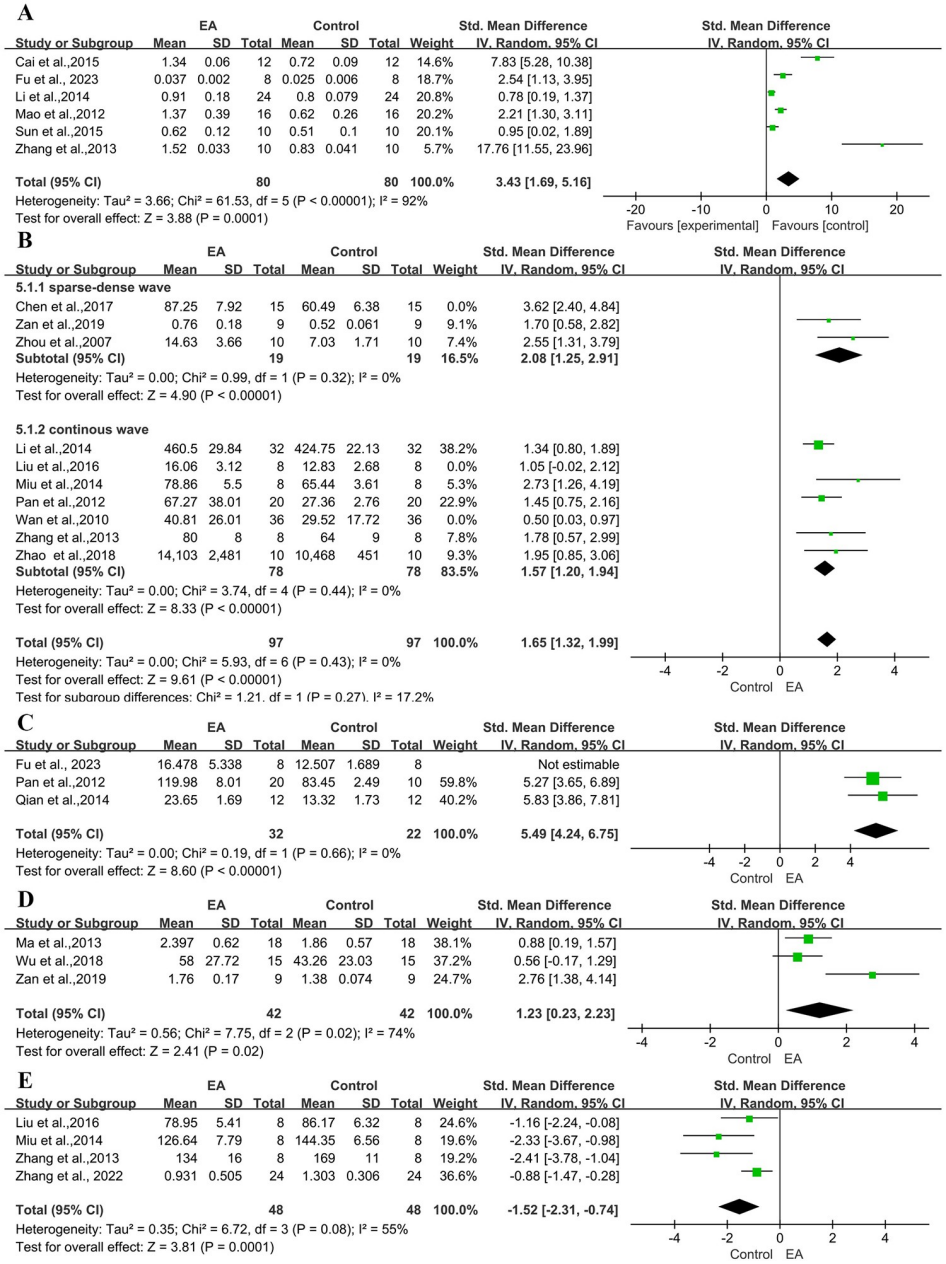
Forest plot of increasing VEGF levels from different studies. (A) PT-RCT, (B) IHC, (C) Serum VEGF content, (D) VEGFR-2 receptor, (E) VEGF average gray value.

*PT-RCT*. Meta-analysis of 6 studies showed a significant difference for improving the mRNA expression of VEGF (Fig 7).

*Immunohistochemistry(IHC)*. A combined analysis of 10 studies showed a significant difference for improving the mRNA expression of VEGF (n = 280, SMD: 1.84, 95% CI 1.21 to 2.47, P < 0.01; Heterogeneity: Chi-square = 34.94, df = 8, P <0.00001, I² = 77%). We performed subgroup analyses based on electroacupuncture stimulation frequency, which showed moderate heterogeneity (Heterogeneity: Sparse-dense Wave: Chi-square = 5.19, df = 2, P = 0.07, I² = 61%; Continous Wave: Chi-square = 16.01, df = 5, P = 0.07, I² = 69%). After two studies [16, 26] were excluded from sensitivity analysis, the remaining studies showed significant differences between electroacupuncture and control. There may be a baseline difference between Wan et al.(2010) [26] and other studies, such as rat body weight (Fig 7).

*Serum VEGF content*. A combined analysis of 3 studies showed the treatment significantly increased serum VEGF levels (Fig 7).

*VEGFR-2 receptor*. A combined analysis of 3 studies showed the treatment promotes the production of VEGFR-2 receptors (Fig 7).

*VEGF average gray value*. A combined analysis of 4 studies showed the treatment reduced the VEGF average gray value (Fig 7).

## 4 Discussion

The current study is the first preclinical meta-analysis of the effects of electroacupuncture targeting the opening of the BBB. Based on strict inclusion and exclusion criteria, 31 articles were adopted, and the quality of the studies was generally moderate. The BBB is responsible for maintaining homeostasis in the brain microenvironment, and a variety of nervous system injuries will damage and destruct it. Several studies have indicated that the release of multiple factors following stroke as well as the change in oxygen pressure following ischemia-reperfusion will destroy oxygen radical absorbance capacity, downregulation of tight junction proteins and upregulation of immunologic factors, resulting in BBB permeability disorders, but drugs continue to be ineffective in crossing the BBB, and the therapeutic effect of central nervous system diseases remains unsatisfactory [44]. A comparative analysis demonstrated that EA can enhance the penetration of EB into the central nervous system and promote the treatment of brain injury with nutritional neurological drugs such as NGF. Its mechanism may be related to the promotion of high levels of VEGF that increase the intercellular space between vascular endothelial cells. Under physiological conditions, EA remains effective at opening the BBB. It is also important to note that different electroacupuncture frequencies have different impact on the results of the BBB opening. As another mechanism for opening the BBB, increasing the expression of MMP-9 and GFAP as well as inhibiting P-gp expression is also utilized.

In addition to mediating angiogenesis, VEGF is an important vascular permeability factor. Reactive AC induces and releases VEGF, alters the distribution of tight junction proteins over the BBB, reduces intravascular and extravascular resistance, and alters the permeability of the BBB as a result. Studies show that VEGF-induced BBB permeability relies on VEGFR2-mediated eNOS pathway downregulation of tight junction expression [39, 40]. Electroacupuncture may increase VEGF mRNA expression, average gray value, and serum VEGF content in animal brains, suggesting that future studies may examine how electroacupuncture affects the VEGF/VEGFR2 signaling pathway to open the blood-brain barrier. Also, CUMS stress reduces the hippocampus’ GFAP-ir protein levels and the mean optical density of astrocytes, a change that can be reversed by EA intervention [34]. GFAP is considered to be a marker for AC activation.

Positive studies have demonstrated that sparse-dense wave electroacupuncture with a frequency of 2/100 Hz can advance the peak of MMP-9 expression in rats after brain infarction [39]. It may be related to the fact that electroacupuncture alters rats’ levels of oxidative stress. The large expression of Matrix Metalloproteinase 9 (MMP-9) can degrade the ECM structure of the BBB, mediate the disintegration of TJ cytoskeleton, and increase the BBB permeability [45, 46]. This study did not provide a systematic summary of electroacupuncture interventions and their effects on TJ protein expression, but previous research has shown that they can affect ZO-1 and occludin expression [8]. Thus, the next step is to examine how electroacupuncture affects TJ protein expression and the oxidative stress signaling pathway.

P-gp uses the energy released during the breakdown of ATP to enable it to efflux exogenous substances from the BBB [47]. P-gp exerts enhanced effect on drug efflux, that is, the barrier effect of the BBB is enhanced. Currently, it has been shown that the TNF-α/PKCβ1/S1PR1 signaling pathway can rapidly reduce the activity of P-gp [48]. EA may directly phosphorylate P-gp by regulating PKCβ1, and it can also control the transcription of the mdr1a gene by phosphorylating transcription factors.

A significant role is played by NGF in MCAO rats. It is currently difficult to cross the blood-brain barrier with the majority of NGFs used clinically due to their large molecule weight of 13KD [49]. The above studies show that electroacupuncture stimulation can facilitate the penetration of drugs such as NGF. The results of this experiment showed that electroacupuncture stimulation could open the blood-brain barrier of healthy rats and rats with central nervous system injury diseases, increase VEGF levels in rats with MCAO, PSD, and VD, and promote EB into the brain of MCAO rats.

Futhermore, different parameters of electroacupuncture stimulation can produce different effects, such as regulating different signaling pathways and protein expression in terms of analgesic mechanisms [50, 51]. Sparse-dense wave therapy had a greater effect on regulating VEGF than continuous wave therapy (sparse-dense wave: SMD: 2.08, continuous wave: SMD: 1.57). However, the effect size measured for 100Hz continuous wave [36] that prompted NGF to enter the brain only once was more significant than for other frequencies. There is no significance in the above subgroup analysis of wave forms, but it is apparent from SMD that dense wave has a greater effect on promoting blood-brain barrier opening after MCAO by increasing VEGF, but at the same time, the 100Hz continuous wave effect is more significant.

It has been shown that various open mechanisms can modulate the state of the BBB in the same way. Acupuncture plus medication can produce pharmacodynamic or pharmacokinetic synergism, reduce neuronal damage, and enhance neuronal survival. Through the use of EA, it is possible to open the BBB so that the dose of medication can be reduced, side effects can be reduced, drug concentrations in the brain can be improved and the treatment index can be improved. In the present time, animal experiments are the main source of basic research on acupuncture, which provides an in-depth understanding of the mechanism of action of acupuncture as well as valuable evidence and reference for clinical experiments [52]. And in future clinical trials, we can utilize multiple mechanisms of action to achieve the optimal strategy for electroacupuncture to open the BBB.

## 5 Limitations

A limitation of this study is that we discussed sparse-dense and continuous waves in terms of electroacupuncture frequency. However, different stimulus frequencies target different targets. As another limitation, most studies used different interventions and extracted data at different times. Therefore, it is difficult to compare the effects of electroacupuncture on the BBB during the acute, chronic, and convalescent phases of a disease. In this study, there were no studies related to the intake of Aquaporin 4 (APQ4), since APQ4 is thought to be an aquaporin, which does not affect the transport of most drugs. Many researches had a moderate to high bias risk, mainly due to a lack of clear reporting of relevant data. The Risk of Bias tool developed by SYRCLE can be incorporated into reports relating to animal experiments to address this issue. In addition, data presented in most studies are graphical, and the lack of raw data may have an impact on the accuracy of the data.

## 6 Conclusion

Based on our findings, EA is capable of opening the BBB, increasing the concentration of macromolecular drugs in the brain, and treating diseases of the central nervous system such as MCAO, PSD, and VD. One of the primary mechanisms of opening the BBB is the stimulation of astrocytes to release VEGF factor, thereby increasing the width of the intercellular space between the vascular endothelial cells. Furthermore, it is also important to regulate MMP-9 and GFAP expression, as well as to inhibit P-gp expression.

## Supporting information

S1 Checklist The PRISMA flow chart illustrates the selection process for systematic reviews. (DOCX)

S1 Fig VEGF. (TIF)
