# Supplementary figures and images for: Electroacupuncture stimulation enhances the permeability of the blood-brain barrier: A systematic review and meta-analysis of preclinical evidence and possible mechanisms
Source: PLoS One. 2024 Mar 27;19(3):e0298533. doi: 10.1371/journal.pone.0298533 (PMC10971611; doi:10.1371/journal.pone.0298533)

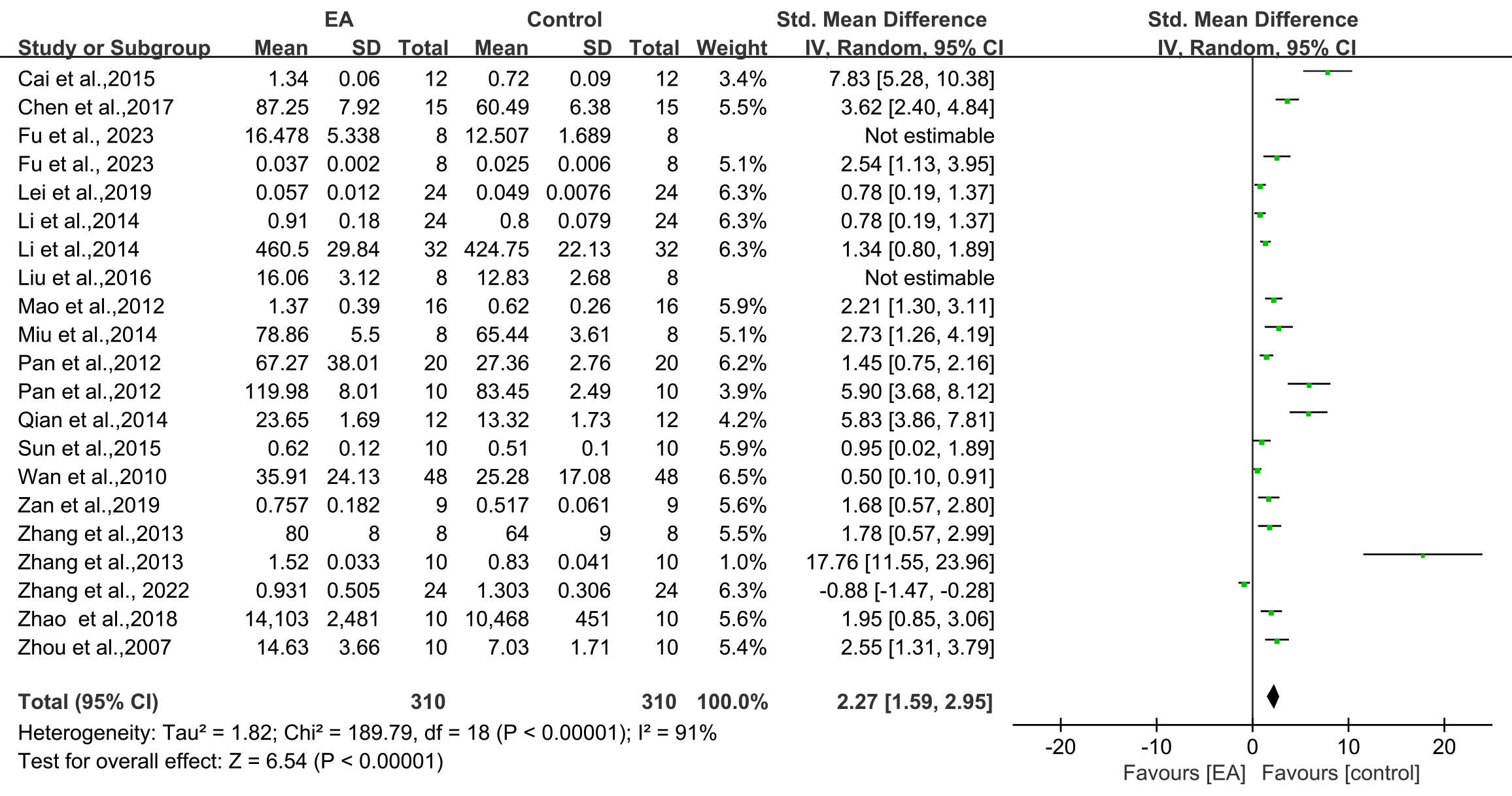

Supplement: S1 Fig — (TIF) [file pone.0298533.s002.tif]
